# Clinical Implications of ESR1 Mutations in Hormone Receptor-Positive Advanced Breast Cancer

**DOI:** 10.3389/fonc.2017.00026

**Published:** 2017-03-15

**Authors:** Tomas Reinert, Everardo D. Saad, Carlos H. Barrios, José Bines

**Affiliations:** ^1^Hospital de Câncer Mãe de Deus, Universidade Federal do Rio Grande do Sul, Porto Alegre, Brazil; ^2^Dendrix Research Ltd., São Paulo, Brazil; ^3^PUCRS School of Medicine, Porto Alegre, Brazil; ^4^Instituto Nacional de Câncer, Rio de Janeiro, Brazil

**Keywords:** breast neoplasms, metastatic disease, endocrine resistance, ESR1, mutations

## Abstract

Hormone receptor-positive breast cancer is the most frequent breast cancer subtype. Endocrine therapy (ET) targeting the estrogen receptor (ER) pathway represents the main initial therapeutic approach. The major strategies include estrogen deprivation and the use of selective estrogen modulators or degraders, which show efficacy in the management of metastatic and early-stage disease. However, clinical resistance associated with progression of disease remains a significant therapeutic challenge. Mutations of the ESR1 gene, which encodes the ER, have been increasingly recognized as an important mechanism of ET resistance, with a prevalence that ranges from 11 to 39%. The majority of these mutations are located within the ligand-binding domain and result in an estrogen-independent constitutive activation of the ER and, therefore, resistance to estrogen deprivation therapy such as aromatase inhibition. ESR1 mutations, most often detected from liquid biopsies, have been consistently associated with a worse outcome and are being currently evaluated as a potential biomarker to guide therapeutic decisions. At the same time, targeted therapy directed to ESR1-mutated clones is an appealing concept with preclinical and clinical work in progress.

## Introduction

Breast cancer is a heterogeneous disease comprising different clinical, histopathological, and molecular subtypes. Hormone receptor-positive (HR+) tumors represent the most common form of breast cancer and account for most of the deaths from the disease. Endocrine therapy (ET) is recommended to virtually all patients with HR+ breast cancer. However, due to a variety of mechanisms of resistance, a significant proportion of patients with early-stage breast cancer experience recurrence despite curative-intent local therapy and long-term adjuvant ET. In the metastatic setting, although most patients derive benefit from initial ET, with disease stabilization or tumor shrinkage, development of resistance invariably occurs (1). Breast tumors are known to undergo genomic evolution during treatment, with the acquisition of new alterations that confer resistance to therapy. ESR1, the gene that encodes the estrogen receptor (ER), is known to undergo ligand-binding domain (LBD) mutations, gene amplification, or an ESR1/YAP1 translocation that are potential mechanisms of resistance to ET (1, 2).

This review summarizes published and ongoing research covering ESR1 mutations in breast cancer, addressing epidemiological, pathophysiological issues with potential clinical implications. We will explore the prospects for analysis of ESR1 mutational status as a prognostic and predictive biomarker as well as the development of therapeutic strategies targeting ESR1-mutated tumor cells.

## The ER Pathway

Estrogen receptor, a protein encoded by the ESR1 gene, is expressed in approximately 70% of breast cancers. ER expression is one of the defining features in classifying tumor subtype and assigning therapeutic strategies in breast cancer. A large body of experimental and clinical research has established the fundamental role of ER and its hormonal ligands in normal mammary gland development and in the etiology and progression of breast cancer (3).

Estrogen receptor is predominantly a nuclear protein that functions as a ligand-dependent transcription factor. Functionally, the ER consists of two transcriptional activation domains: the N-terminal, ligand-independent activation function domain, and the C-terminal, ligand-dependent AF-2 domain. The LBD resides in the C-terminal region, while the DNA-binding and hinge domains are positioned in the central core of the protein (2). Estrogen binding triggers a number of events resulting in activation of ER and induces conformational changes in the LBD, allowing the estrogen–ER complex to bind to specific DNA sequences [estrogen response elements (EREs)] while interacting with co-repressor and coactivator proteins to regulate the transcription of estrogen-responsive genes that are important in various physiological and pathological processes, including carcinogenesis and tumor progression (2, 4–6).

## ET for Advanced Breast Cancer: Where are We?

Targeting the ER pathway with endocrine therapies may be considered the first molecularly targeted treatment for cancer and remains a mainstay of treatment for all stages of ER-positive disease (7). The main strategies of ET include treatments that result in estrogen deprivation [e.g., ovarian ablation, aromatase inhibitors (AIs)], drugs that antagonize the ER (e.g., tamoxifen), and ER downregulation (e.g., fulvestrant). Endocrine agents are used throughout all stages of the breast cancer clinical continuum. Endocrine monotherapy is standard of care as a chemoprevention strategy in patients with *in situ* tumors, adjuvant treatment for patients with early-stage disease, and as treatment aiming at disease control and survival prolongation for patients with metastatic disease. While the benefits of ET are clearly recognized, unfortunately, breast tumors are known to undergo genomic evolution, with the acquisition of new alterations that confer resistance to therapy. Therefore, a significant proportion of patients with early-stage breast cancer experience recurrence despite local therapy with curative intent and long-term adjuvant ET. In the metastatic setting, although most patients derive benefit from initial ET, with disease stabilization or tumor shrinkage, subsequent lines of treatment result in shorter periods of response, denoting the development of resistance and disease progression that invariably occurs (1).

Recent developments in the understanding of molecular interactions between ER signaling and growth factor, metabolic and cell-division pathways have opened the possibility of improving results by modulating hormone signaling and interfering with resistance mechanisms yet to be fully understood (7). As a result of some of these developments, the treatment algorithm for HR+ advanced breast cancer is evolving, and combinations of endocrine agents with targeted therapies that modulate endocrine resistance, such as mTOR and CDK 4/6 inhibitors, have been recently incorporated into clinical practice and are covered in different guidelines (8, 9). Major paradigms that have been guiding clinical practice include the sequential use of endocrine agents and the indication of ET in all cases, except those with impending visceral crisis or proven endocrine resistance. Primary endocrine resistance has been arbitrarily defined as a relapse while on the first 2 years of adjuvant ET, or PD within the first 6 months of first-line ET for metastatic disease. Secondary (acquired) endocrine resistance has been defined as a relapse while on adjuvant ET but after the first 2 years, or a relapse within 12 months of completing adjuvant ET, or as disease progression that occurs ≥6 months after initiating ET for metastatic disease (7, 10). However, inter- and intra-tumor heterogeneity (11), combined with limitations in the design of trials conducted in this area, and the absence of predictive biomarkers make it difficult to develop a more rational approach for HR+ advanced breast cancer and to define the optimal sequencing of endocrine agents and whether endocrine therapies should be used in combination or sequence with targeted therapies (7, 8). Clinical recommendations about ET for women with HR+ advanced breast cancer have been comprehensively reviewed in a recent American Society of Clinical Oncology Clinical Practice Guideline (8).

## ESR1 Mutations

ESR1 mutations were first described in cell models in 1996 (12), when Y537S and E380Q mutations were found to confer constitutive activation of the receptor and resistance to endocrine agents. Shortly thereafter, the Y537N mutation was found in a clinical sample of metastatic breast cancer (13). However, subsequent studies performed mainly in primary breast tumors were not able to identify ESR1 mutations, and the potential clinical significance of the abnormality remained underappreciated for more than a decade. Large-scale genomics efforts, such as The Cancer Genome Atlas (TCGA) project, have led to new insights in the landscape and complexity of breast cancer genomics and heterogeneity (14). Despite the central role of the ER in luminal tumors, TCGA data for 962 breast cancer samples indicated that ESR1 mutations were present in only 0.5% of primary breast tumor cases (15).

It was not until 2013 that a series of studies using next-generation sequencing (NGS) of DNA renewed interest in the mutated receptor by demonstrating a high prevalence (11–55%) of ESR1 mutations in metastatic ER-positive breast cancers with prior AI exposure (2, 16–20). In a review by Jeselsohn et al., a total of 187 metastatic ER+ breast tumors from patients in five studies treated with at least one line of ET were described: ER LBD mutations were identified in 39 cases (21%) (2). In other published series, the prevalence of ESR1 mutations has varied from 11 to 54%, mostly because of differences in patient profiles (see Table 1). Most recent studies analyzing ESR1 mutations in liquid biopsies in cohorts of clinical trials in AI-refractory HR+ advanced breast cancer suggest a prevalence ranging from 11 to 39% (21–26). In contrast, ESR1 mutations have been rarely detected in treatment-naïve primary tumors, suggesting that these mutations arise either through clonal selection of very low abundance resistant clones or are acquired later in the disease course under the selective pressure of ET.

**Table 1:** **Prevalence of ESR1m in published studies including more than 100 patients**.

Reference	Number of patients (*n*)	ESR1m prevalence (%)	Methods
Fribbens et al. (PALOMA 3 cohort) (23)	360	25	Plasma circulating tumor DNA (ctDNA) ddPCR
Fribbens et al. (SOFEA cohort) (23)	161	39	Plasma ctDNA ddPCR
Chandarlapaty et al. (BOLERO-2 cohort) (21)	541	29	Plasma ctDNA ddPCR
Clatot (22)	144	31	Plasma ctDNA ddPCR
Spoerke (FERGI cohort) (25)	153	37	Plasma ctDNA ddPCR
Schiavon (24)	171	11	Plasma ctDNA ddPCR
Jeselson (2)	187	21	Metastatic tumor biopsies next-generation sequencing (NGS)
Niu (26)	222	12	Metastatic tumor biopsies NGS

**Figure 1:**
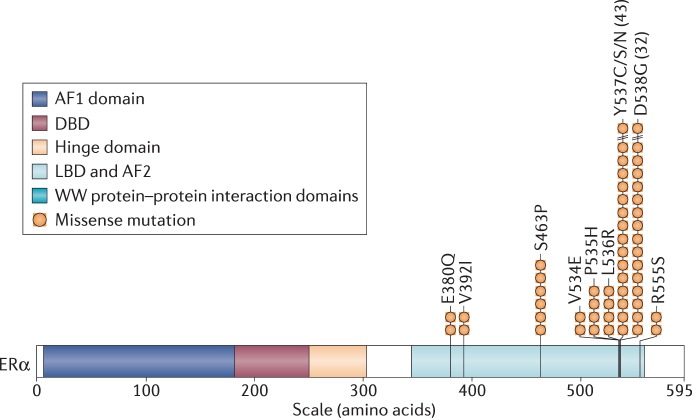
**The ESR1 gene and most common mutations [reprinted with permission—Ma et al. (1)]**. A schematic diagram of estrogen receptor-α (ERα) mutations and their frequencies in ER+ metastatic breast cancer after therapy with aromatase inhibitors and other endocrine agents. The structural domains of ERα are shown, including the transcription activation function 1 (AF1) domain, the DNA-binding domain (DBD), the receptor dimerization and nuclear localization (hinge) domain, and the ligand-binding domain (LBD), and AF2 domain.

ESR1 mutations are most commonly missense mutations clustered in codons 537 and 538 of the LBD that result in ligand-independent constitutive activation of the receptor. The most prevalent ESR1 point mutations are Y537S and D538G, while several others have been identified at significantly lower frequencies. Remarkably, the majority of ESR1 mutations localize to just a few amino acids within or near the critical helix 12 region of the ER LBD, where they are likely to be single-allele mutations, as shown in Figure 1 (1, 27). Mutant ER recruits coactivators in the absence of hormone, while their affinities for estrogen agonist (estradiol) and antagonist (4-hydroxytamoxifen) are reduced. Further, they confer antiestrogen resistance by altering the conformational dynamics of the loop connecting Helix 11 and Helix 12 in the LBD of ER, which leads to a stabilized agonist state and an altered antagonist state that resists inhibition (28).

## Subgroups at Increased Risk of Developing ESR1 Mutations

Based on available data, it could be hypothesized that some factors such as previous ET exposure and patient and disease characteristics could be associated with an increased risk of ESR1 mutations within patients with ER+ advanced breast cancer.

### Exposure to Previous Endocrine Therapies

From a mechanistic point of view, two general patterns of ET resistance may be recognized clinically: primary (or intrinsic) resistance, whereby ER+ cancers never adequately respond to ET, and secondary (or acquired) resistance, which develops following an initial response (1, 29). These definitions, although imperfect and arbitrary, have been useful in some clinical trials to stratify patient populations (30). As noted above, ESR1 mutations are characteristically absent in primary tumors and are an unlikely mechanism of primary resistance (2, 15). Most patients with tumors harboring ESR1 mutations seem to experience a protracted clinical course prior to detection of the mutation, supporting the idea that this is largely an acquired (secondary) resistance mechanism that emerges after long-term ET (31). Several studies have shown an association between the prevalence of the hot spot LBD mutations and the number of lines of ET (16). Interestingly, kinetic studies showed detectable circulating ESR1 mutations before clinical progression in 75% of the cases (22). If further confirmed and validated that these results may eventually justify earlier changes in treatment strategies based on liquid biopsies, before radiographic or clinical demonstration of disease progression.

### Tamoxifen versus AIs

Based on current evidence, ESR1 mutations are chiefly a mechanism of resistance to AIs rather than a non-specific mechanism of resistance to endocrine agents in general. ESR1 mutations have been identified only rarely in patients whose sole previous ET was tamoxifen (16, 23, 24). Although much of our understanding of ligand-independent ER activity comes from analysis of tamoxifen resistance, it must be stressed that while tamoxifen and AI resistance share many common features, their effects on ER signaling are far from identical.

### Distant versus Locoregional Disease

Early studies reported ESR1 mutations in tumor samples obtained from different organ sites, including lymph nodes, skin, lung, and liver, suggesting that these mutations do not display specific organotropism (2, 16–18, 20). In contrast, recently published multivariable analyses based on circulating DNA of patients from the SOFEA and PALOMA3 trials showed that the detection of ESR1 mutations is associated with bone and visceral disease and may suggest that ESR1 mutations are unfrequently detected in patients with nodal or loco regional recurrence only (23). These associations should be evaluated in further studies.

### AI Exposure on the Adjuvant versus on the Metastatic Setting

Some studies suggest that the prevalence of ESR1 mutations differs markedly between patients who were first exposed to AI during adjuvant therapy and those exposed in the metastatic setting (21, 24). ESR1 mutations are rarely acquired during adjuvant AI (21, 24) but are commonly detected after therapy for metastatic disease, suggesting that mechanisms of resistance to targeted therapy may be substantially different between the treatment of micrometastatic and overt metastatic cancer. Schiavon et al. explained this difference by hypothesizing that preexisting ESR1 mutant subclones are selected by AI therapy, but the tumor burden in the micrometastatic setting may be too low for such clones to be detected (24). This hypothesis-generating association should be further evaluated in prospective studies, even more so with the recent publication of the extension of AI adjuvant therapy to 10 years (32).

**Which subgroups are at higher risk of developing ESR1m?**

– HR+ advanced breast cancer
– Resistance to AIs
– Secondary (acquired) resistance
– Long disease control interval with ET
– Aromatase inhibitors used in the metastatic setting
– Bone and visceral disease

## How Should We Test for ESR1 Mutations?

Initial studies describing ESR1 mutations were performed in metastatic tumor samples from retrospective cohorts and clinical trials. Mutations were detected by whole-genome sequencing and were confirmed with analysis of the originating tumors (2). With the potential evolution of the tumor genome through treatment, repeated sampling of a tumor would be required to optimally guide therapy, because the mechanism of resistance may not be evident in analyses of pre-treatment samples. Yet, serial biopsies of recurrent, metastatic cancer would be invasive, risky, and unacceptable to many patients.

The ability to study non-hematologic cancers through minimally invasive sampling of blood is an exciting and rapidly advancing field in cancer diagnostics. These liquid biopsies have been driven both by major technologic advances, including the isolation of intact cancer cells and the analysis of cancer cell-derived DNA from blood samples and by the increasing application of molecularly driven therapeutics, which rely on accurate and timely measurements of biomarkers (33). Tumor-derived DNA is found in the plasma of patients with recurrent cancer, and in-depth analysis of circulating tumor DNA (ctDNA) presents a non-invasive way of analyzing tumor genetics and the acquisition of selected abnormalities throughout the course of treatment. Numerous recent reports have demonstrated the detection of mutant ESR1 DNA alleles as tumor-specific biomarkers in cell-free DNA (cfDNA) from blood (21, 23, 24, 34–37). In this context, digital polymerase chain reaction (PCR)-based methods appear to be a simpler and more sensitive approach for *ESR1* detection in ctDNA than NGS techniques (35, 36, 38).

## ESR1 Mutation as a Biomarker

At the present time, there is no evidence demonstrating a role for specific biomarkers other than ER, PR, and HER2 in the clinical management of HR+ advanced breast cancer (7, 8). Use of other biomarkers is considered experimental and currently should be reserved for selection of treatment in clinical trials. Technical developments in sequencing ctDNA, among other ongoing efforts attempting to define changes induced by previous treatments, will allow us to better understand what happens after HR signaling is altered by therapy. The availability of new sensitive sequencing technologies to analyze data from prospective and retrospective studies will provide important information on the clinical significance of ESR1 mutations and possibly guide the development of novel therapeutic strategies.

## Prognostic Biomarker

ESR1 mutations have been consistently associated with statistically and clinically inferior outcomes in several series. Worst prognosis has been demonstrated as decreased progression-free survival (PFS) and overall survival (OS), in comparison with patients with wild-type ESR1.

Chandarlapaty et al. (21) analyzed cfDNA from baseline plasma samples from participants in the BOLERO-2 trial. The two most frequent mutations in *ESR1* (Y537S and D538G) were analyzed and samples were scored as wild-type, D538G, Y537S, or double-mutant. Cox proportional hazards model was used to assess PFS and OS in patient subgroups defined by mutations. Of 541 evaluable patients, 156 (28.8%) had one or both *ESR1* mutations (D538G in 21.1% and Y537S in 13.3%, with 30 patients having both). These mutations were associated with shorter OS (wild-type, median of 32 months; D538G, median of 26 months; Y537S, median of 20 months; both mutations, median of 15 months, supporting the notion that ESR1 mutations are an adverse prognostic biomarker associated with more aggressive disease biology). The adverse prognostic impact of ESR1 mutations remained after adjustment for the potential effects of previous hormone therapy, visceral disease, and performance status.

Similarly, Clatot et al. (22) reported a retrospective analysis of predictive and prognostic values of ESR1 circulating mutations (D538G and Y537S/N/C) in advanced breast cancer after progression on AI treatment. Among the 141 patients analyzed, the median OS was significantly shorter in patients with circulating *ESR1* mutation (15.5 months) than in patients without mutations (23.8 months; *P* = 0.0006; hazard ratio = 1.9). The prognostic value of circulating *ESR1* mutations at progression was confirmed in multivariable analysis (*P* = 0.002, hazard ratio = 1.9). A level of cfDNA above the median value and a performance status >1 were also identified as independent prognostic factors for OS. A worse PFS was observed in patients with *ESR1* mutations, with a median of 5.9 months, compared with 7.0 months for patients without mutations (*P* = 0.002, hazard ratio = 1.7). In the multivariable analysis of PFS, the presence of circulating *ESR1* mutation and a prior line of chemotherapy before AI introduction were identified as independent prognostic factors of worse outcome.

## Predictive Biomarker

As mentioned before, there is no evidence to date demonstrating a role for a biomarker in selecting an optimal therapeutic strategy for ER-positive HER2-negative advanced breast cancer. Limitations associated with tumor heterogeneity and caveats in the design of trials conducted in this area have made it difficult to develop predictive biomarkers, and most of the new combinations with targeted agents, even though showing improvements in clinical endpoints, have been directed to unselected populations of patients (39–42). However, recent data from several studies published over the last 2 years have brought interest in the potential role of ESR1 mutational status as a predictive biomarker and a tool to guide the clinician in therapeutic decisions. The ESR1 mutation behavior has also been described in small series: although most patients with an increase in circulating ESR1 mutation had disease progression, not all patients with a decrease in ESR1 mutation responded to treatment (22, 37). Available data on the use of ESR1 mutation as predictive biomarkers for different endocrine agents are summarized in the following paragraphs.

### Aromatase Inhibitors

Aromatase inhibitors inhibit breast cancer growth by estrogen deprivation and, therefore, ESR1 mutations confer resistance to aromatase inhibition because they allow tumors to proliferate independently of estrogen. Patients with ESR1 mutations have a poorer PFS on subsequent AI-based therapy than patients without such mutations (23, 24). In a prospective–retrospective analysis of the SOFEA trial (43), the detection of ESR1 mutations in plasma DNA predicted relative resistance to exemestane (23). This provides initial evidence of the potential clinical utility for the use of ESR1 plasma DNA analysis in selecting the most appropriate ET.

### Tamoxifen and Fulvestrant

Preclinical studies on the effect of ER-LBD mutations have shown a relative resistance of the activating mutations to tamoxifen and fulvestrant (2, 13). On the other hand, effective inhibition of these mutants with higher doses of these agents suggests that the use of such higher doses or of more-potent (or mutant-specific) selective ER modulators (SERMs) or selective ER downregulators (SERDs) might benefit patients with tumors harboring LBD-mutated ER. In a retrospective analysis of patients treated with 500 mg of fulvestrant as monotherapy in the FERGI trial, ESR1 mutations were not associated with a differential PFS benefit, suggesting that they may not be strongly associated with clinical resistance to SERD treatment (25). In a prospective–retrospective analysis, Fribbens et al. (23) assessed ESR1mutations in available archived baseline plasma from the SOFEA trial, which compared exemestane with fulvestrant-containing regimens in patients with prior sensitivity to non-steroidal AI, and in baseline plasma from the PALOMA3 trial, which compared fulvestrant plus placebo versus fulvestrant plus palbociclib in patients with progression after receiving prior ET. ESR1 mutations were analyzed by multiplex digital PCR. The results suggest that ESR1-mutant cancers show selective sensitivity to fulvestrant, a drug that degrades the ER, in comparison to AIs. Nonetheless, ESR1 mutants treated with fulvestrant had modestly worse PFS than wild-type cancers. This is consistent with the finding that *in vitro* hot spot mutations in the LBD partially inhibit fulvestrant binding (18). More potent receptor degraders may have the potential to further improve results in comparison with fulvestrant in ESR1 mutant cancers, and a number of such therapies are in early clinical development.

### Chemotherapy

The impact of ESR1 mutational status on chemotherapy has not been adequately studied. A French cohort evaluated 74 patients and also reported worse outcomes for ESR1-mutated patients irrespective of treatment with ET or chemotherapy. No particular benefit of chemotherapy versus tamoxifen or fulvestrant was seen (44).

### mTOR Inhibitors

The combination of mTOR inhibitors with endocrine agents has been incorporated into clinical practice after the publication of the BOLERO 2 trial, a randomized phase III study that demonstrated significant improvement in PFS with the addition of the mTOR inhibitor everolimus to the steroidal AI exemestane (39) in patients with HR+ advanced breast cancer that progressed after non-steroidal AI therapy. As noted above, Chandarlapaty et al. used liquid biopsies of baseline plasma samples of patients participating in the BOLERO2 trial (21) to assess the potential role of ESR1 mutations as prognostic and predictive biomarkers. Both Y537S and D538G mutations were associated with more aggressive disease biology and patients harboring ESR1 mutations had worst prognosis with shorter OS. With respect to the benefit of everolimus, the analysis confirmed that most patients benefit from the addition of this drug since both the wild-type and mutant groups had a demonstrated increase in PFS from the addition of everolimus. By mutation site, the benefit was evident for the D538G subgroup. The study lacked sufficient numbers of patients with Y537S to draw conclusions on everolimus benefit for this subgroup. An interaction between specific alleles and mTOR activity has not been biologically identified. Therefore, this particular data are hypothesis generating and further biological and clinical investigation into potential ESR1 mutant allele-specific effects should be encouraged.

### CDK4/6 Inhibitors

The addition of CDK4/6 inhibitors to ET is one of the major breakthroughs in breast oncology over the last few years. Palbociclib is currently used in routine clinical practice in the USA both in first- and second-line setting following its approval by the Food and Drug Administration based on three randomized clinical trials (PALOMA 1, 2, and 3) (40, 41, 45) that demonstrated a significant benefit in terms of PFS and clinical benefit rate with its addition to endocrine agents.

The PALOMA3 trial evaluated the combination of palbociclib with fulvestrant in AI-resistant patients (41). The ESR1 mutation status was analyzed in 69% of all patients, with ESR1 mutations detected in 25.3% of cases (23). There was a predominance of mutations in D538G, Y537N, Y537S, and E380Q. Mutations were polyclonal in 28.6% of cases. Characteristics associated with ESR1 mutation were sensitivity to prior ET, bone metastases, and prior lines of therapy for metastatic disease. The benefit from palbociclib was seen irrespective of ESR1 mutation status.

Many patients with metastatic ER-positive HER2-negative breast cancer now receive letrozole and palbociclib as first-line ET (46). Although Fribbens et al. (23) showed encouraging activity of both fulvestrant and palbociclib against ESR1-mutant cancers, it is not known how palbociclib will affect the emergence of ESR1 mutations or whether fulvestrant plus palbociclib will have the same benefit in patients with prior palbociclib exposure (47). A recent report suggests that treatment with palbociclib and letrozol does not prevent selection of ESR1 mutations (23).

Data from clinical trials demonstrating clinical benefits have been reported for two other inhibitors of CDK4 and CDK6 (namely, ribociclib and abemaciclib) (42, 48). At the present time, no data on the potential interaction of ESR1 mutations with these targeted agents have been reported.

## ESR1 Mutation as a Therapeutic Target

Targeted therapy directed to ESR1-mutated clones is an appealing concept, and preclinical and clinical development of rationale-based novel therapeutic strategies that inhibit these ER mutants has the potential to substantially improve treatment outcomes. New-generation SERMs and SERDs, such as bazedoxifene (49) and ARN 810 (50), are being studied in initial phase I/II clinical trials after preclinical evidence of efficacy in the inhibition of ER-LBD mutants (2, 51). AZD9496, a non-steroidal small molecule, which is a potent and selective antagonist and downregulator of ERα can potently bind and downregulate D538G and Y537C/N/S ERα proteins *in vitro* (52).

The activity of mutated ER remains highly dependent on the recruitment of coactivators; therefore, new agents targeting ER coactivators, such as small-molecule SRC-3 inhibitors, might offer another approach to target the ER mutants and should be tested alone and in combination with ER antagonists (53).

The presence of ESR1 mutations is associated with other genetic and epigenetic alterations; therefore, testing of novel therapeutic agents and their combinations in preclinical models that include different genetic backgrounds is crucial and highlights the potential of patient-derived xenografts and *ex vivo* cultures of circulating tumor cells (CTCs) from patients with metastatic ER+ breast cancer.

Indeed, a recent report successfully used *ex vivo* cultures of CTCs harboring ER mutants to tailor different therapeutic combinations. Yu et al. (54) tested inhibitors of multiple pathways, alone or in combination with ER inhibitors. The results were highly complex and varied between the two available models, but the study identified the efficacy of mTOR, PI3K, and heat shock protein 90 (HSP90) inhibitors in this setting, especially in combination with fulvestrant. Targeting HSP 90, which is the chaperone protein of ER, may be useful to treat Y537S ESR1-mutated tumors. The authors showed that mutant ESR1 tumors are highly dependent on HSP90 and preclinical studies with the HSP90 inhibitor STA9090 demonstrated cytotoxicity alone and in combination with raloxifene and fulvestrant to *ex vivo* cultured circulating breast tumor cells (54). Interestingly, they also described that the allele frequency of ESR1 mutation correlated with the sensitivity to HSP90 inhibition.

On the basis of the available studies, new therapeutic strategies should first be studied in preclinical models that accurately demonstrate the genomic complexity of individual tumors. If successfully translated to the clinical setting, our abilities to better detect the mutations, predict resistance, and effectively treat tumor harboring these mechanisms of resistance will have an important impact on patient outcomes.

## Future Perspectives

A strategy for individualized, biomarker-driven selection of targeted agents and an integrated method for detecting reproducible key molecular alterations, which cause resistance to ET, are mandatory for future precision medicine in this subset of breast cancer (55).

Alternative approaches enabling the detection of the mutation in liquid biopsies, including the analysis of cfDNA from blood samples, along with additional ultrasensitive methods are in development. Methodological assays to detect and quantify ESR1 mutations in blood and tissue must now be standardized before potential clinical use. It should be noted that there may be other mutations or aberrations in ESR1, such as amplification or translocations, which could also contribute to AI resistance (1). ESR1 mutations are generally clustered between amino acids 534–538, though mutations at other positions, including S463 and E380, have also been described (1, 2, 25). Four hot spot mutations (D538G, Y537S/N/C) contribute to approximately three-quarters of all *ESR1* acquired mutations. The question of whether there are differences between specific mutations is still unanswered. Interestingly, retrospective analysis from the BOLERO-2 trial suggest that the benefit of mTOR targeted therapy was seen in patients with wild-type ESR1 and ESR1 mutations on codon 538, whereas preliminary data showed a potential lack of benefit with everolimus in patients with codon 573 mutations (21). These associations should be evaluated in further studies.

Although the technical sensitivity of the various approaches for mutation detection is known, the level of ctDNA in early-stage patients, among different tumor types, or in various clinical scenarios has not been characterized. Recent data will now support studies on earlier stages of disease. For example, will detection of ESR1 mutations during adjuvant AI therapy affect decisions regarding therapy duration and/or a switch to an alternative therapy?

Neoadjuvant ET (NET) is being increasingly studied both as a clinical and as a scientific tool (56, 57). The evaluation of treatment response *in vivo* is of the highest importance, especially when the disease is still within a “window of curability” (1). Miller et al. (58) recently demonstrated that many mutations are newly detected or enriched post-NET, including two LBD mutation in ESR1 in patients treated with neoadjuvant AI, suggesting that ESR1 mutations may be potential mechanisms of resistance to ET used in early-stage disease and confirming the notion that precision medicine approaches based on genomic analysis of a single specimen are likely insufficient to capture all clinically significant information.

Future research will address whether analyses based on liquid biopsies can be used as a surrogate for treatment response and to monitor disease evolution (59). Another potential use is disease monitoring in metastatic patients and early detection of progression. As mentioned, mutations were detectable in 75% of all cases at least 3 months before progression on AI therapy (22). Future clinical trials in advanced breast cancer might consider using plasma DNA analysis to optimize ET choice according to ESR1 mutation status. An additional open question is whether and how more effective endocrine therapies or longer durations of adjuvant treatment, by increasing the selection pressure and its time-span, will affect the time of emergence and frequency of ESR1 mutations.

Translational research strategies such as PDXs and *ex vivo* cultures of CTCs can be used to facilitate the development of ESR1-mutant targeted therapies. New therapeutic strategies should initially be studied in preclinical models that accurately recapitulate the genomic complexity of breast cancer. If successfully translated to the clinical setting, our abilities to better detect the molecular alterations, predict resistance, and effectively treat tumors harboring these mutations will have a substantial impact on patient outcome.

**Take home messages**

– Recurrent activating mutations within the ER LBD have been detected in 20–30% of patients with metastatic ER-positive endocrine-resistant advanced breast cancer
– ESR1 mutations (ESR1m) confer constitutive ligand-independent activity
– Mechanism of secondary (acquired) resistance to AIs
– Negative prognostic biomarker (inferior survival outcomes)
– May be amendable for monitoring tumor relapse (and earlier treatment change?)
– Potential use as predictive biomarker (resistance to further AI monotherapy, similar/benefit with fulvestrant HD and combination with CDK4/6 and mTOR inhibitors)
– Potential use as therapeutic target (new generations SERMs and SERDs, ER coactivator inhibitors)

## Conclusion

Hormone receptor-positive advanced breast cancer represents a significant clinical problem that is responsible for most breast cancer deaths. Historically, ET has been an effective therapeutic approach associated with both efficacy and limited toxicity. However, our clinical approach to these patients has significant limitations. We tend to treat HR+ patients as having the same endocrine sensitivity, not considering tumor heterogeneity or changes associated with disease progression.

There is an unmet need to generate predictive biomarkers to guide more personalized care for these patients. The discovery of recurrent ESR1 mutations within the region of the gene that encodes the ER-LBD in endocrine-resistant ER+ advanced breast cancer introduced new clinical challenges and opportunities for the understanding of the mechanisms of endocrine resistance. What is clear is that AI-treated tumor cells are adapting to the lack of ligand, and a key aspect to understanding AI resistance is to sample recurrent tumor cells and monitor the signaling pathways that have become active rather than relying purely on the prognostic information available from the treatment-naïve primary tumor.

At the present time, ESR1 mutation status can be considered a negative prognostic biomarker associated with worsened PFS and OS. The potential use of ESR1 mutation status as a predictive biomarker to guide the choice of the optimal therapeutic strategy is being developed rapidly. Based on current data, ESR1 mutations are associated with inferior outcomes with further AI treatment. Benefits with high-dose fulvestrant and combinations of endocrine agents with targeted therapies (CDK4/6 inhibitors and mTOR inhibitors) were seen irrespective of ESR1 mutation status. Targeting ESR1-mutated clones is an appealing concept, and preclinical and clinical development of new-generation SERDS and SERMS as well as ER coactivator inhibitors is ongoing.

Integrative approaches using multiple types of data, such as more comprehensive analysis of the transcriptome, epigenetic regulators of the genome and modern quantitative proteomics methods, coupled with a conceptual bioinformatics and statistical methods that incorporate the intra-tumor genetic and phenotypic heterogeneity found in cancers, may result in fundamental developments that could potentially be translated into clinical benefit for cancer patients (60). Future research efforts should allow ESR1 mutational status to be used as an integral biomarker in trials on ER-positive breast cancer and to be tested prospectively as a stratification factor, as an enrichment strategy and as a therapeutic target in the development of new strategies to overcome endocrine resistance in breast cancer.

## Author Contributions

Conception and design; collection and assembly of data; data analysis and interpretation: TR, ES, CB, and JB.

## Conflict of Interest Statement

The authors declare that the research was conducted in the absence of any commercial or financial relationships that could be construed as a potential conflict of interest.

## References

[1] Ma C Reinert T Chmielewska I Ellis M Mechanisms of aromatase inhibitors resistance. Nat Rev Cancer (2015) 15:261–75. 10.1038/nrc3920 25907219

[2] Jeselsohn R Buchwalter G De Angelis C Brown M Schiff R ESR1 mutations – a mechanism for acquired endocrine resistance in breast cancer. Nat Rev Clin Oncol (2015) 12:573–83. 10.1038/nrclinonc.2015.117 26122181 PMC4911210

[3] Huang B Warner M Gustafsson J Estrogen receptors in breast carcinogenesis and endocrine therapy. Mol Cell Endocrinol (2015) 418:240–4. 10.1016/j.mce.2014.11.015 25433206

[4] Osborne CK Schiff R Mechanisms of endocrine resistance in breast cancer. Annu Rev Med (2011) 62:233–47. 10.1146/annurev-med-070909-182917 20887199 PMC3656649

[5] Schiff R Massarweh SA Shou J Bharwani L Mohsin SK Osborne CK. Cross-talk between estrogen receptor and growth factor pathways as a molecular target for overcoming endocrine resistance. Clin Cancer Res (2004) 10:331S–6S. 10.1158/1078-0432.CCR-031212 14734488

[6] Schiff R Advanced concepts in oestrogen receptor biology and breast cancer endocrine resistance: implicated role of growth factor signalling and oestrogen receptor coregulators. Cancer Chemother Pharmacol (2005) 56:10–20. 10.1007/s00280-005-0108-2 16273359

[7] Reinert T Barrios C. Optimal management of hormone receptor positive metastatic breast cancer in 2016. Ther Adv Med Oncol (2015) 7:304–20. 10.1177/1758834015608993 26557899 PMC4622303

[8] Rugo HS Rumble RB Macrae E Barton DL Connoly HK Dickler MN Endocrine therapy for hormone receptor-positive metastatic breast cancer: American Society of Clinical Oncology Guideline. J Clin Oncol (2016) 34(25):3069–103. 10.1200/JCO.2016.67.1487 27217461

[9] NCCN CLinical Practice Guidelines in Oncology. Breast Cancer. Version 2.2016. Available from: https://www.nccn.org/professionals/physician_gls/pdf/breast.pdf

[10] Cardoso F Costa A Norton I Senkus E Aapro M André F ESO-ESMO 2nd international consensus guidelines for advanced breast cancer (ABC2). Breast (2014) 23:489–502. 10.1016/j.breast.2014.08.009 25244983

[11] Zardavas D Irrthum A Swanton C Piccart M. Clinical management of breast cancer heterogeneity. Nat Rev Clin Oncol (2015) 12:381–94. 10.1038/nrclinonc.2015.73 25895611

[12] Weis KE Ekena K Thomas JA Lazennec G Katzenellenbogen BS. Constitutively active human estrogen receptors containing amino acid substitutions for tyrosine 537 in the receptor protein. Mol Endocrinol (1996) 10:1388–98. 10.1210/mend.10.11.8923465 8923465

[13] Zhang QX Borg A Wolf DM Oesterreich S Fuqua SA. An estrogen receptor mutant with strong hormone-independent activity from a metastatic breast cancer. Cancer Res (1997) 57:1244–9. 9102207

[14] Ma CX Ellis MJ. The Cancer Genome Atlas: clinical applications for breast cancer. Oncology (Williston Park) (2013) 27:1263–9. 24624545

[15] Cancer Genome Atlas Network. Comprehensive molecular portraits of human breast tumours. Nature (2012) 490:61–70. 10.1038/nature11412 23000897 PMC3465532

[16] Jeselsohn R Yelensky R Buchwalter G Frampton G Meric-Bernstam F Gonzalez-Angulo AM Emergence of constitutively active estrogen receptor-α mutations in pretreated advanced estrogen receptor-positive breast cancer. Clin Cancer Res (2014) 20:1757–67. 10.1158/1078-0432.CCR-13-2332 24398047 PMC3998833

[17] Merenbakh-Lamin K Ben-Baruch N Yeheskel A Dvir A Soussan-Gutman L Jeselsohn R D538G mutation in estrogen receptor-α: a novel mechanism for acquired endocrine resistance in breast cancer. Cancer Res (2013) 73:6856–64. 10.1158/0008-5472.CAN-13-1197 24217577

[18] Toy W Shen Y Won H Green B Sakr RA Will M ESR1 ligand-binding domain mutations in hormone-resistant breast cancer. Nat Genet (2013) 45:1439–45. 10.1038/ng.2822 24185512 PMC3903423

[19] Robinson DR Wu YM Vats P Su F Lonigro RJ Cao X Activating ESR1 mutations in hormone-resistant metastatic breast cancer. Nat Genet (2013) 45:1446–51. 10.1038/ng.2823 24185510 PMC4009946

[20] Li S Shen D Shao J Crowder R Liu W Prat A Endocrine-therapy-resistant ESR1 variants revealed by genomic characterization of breast-cancer-derived xenografts. Cell Rep (2013) 4:1116–30. 10.1016/j.celrep.2013.08.022 24055055 PMC3881975

[21] Chandarlapaty S Chen D He W Sung P Samoila A You D Prevalence of ESR1 mutations in cell-free DNA and outcomes in metastatic breast cancer: a secondary analysis of the BOLERO-2 clinical trial. JAMA Oncol (2016) 2:1310–5. 10.1001/jamaoncol.2016.1279 27532364 PMC5063698

[22] Clatot F Perdrix A Augusto L Beaussire L Delacour J Calbrix C Kinetics, prognostic and predictive values of ESR1 circulating mutations in metastatic breast cancer patients progressing on aromatase inhibitor. Oncotarget (2016) 7(46):74448–59. 10.18632/oncotarget.12950 27801670 PMC5342678

[23] Fribbens C O’Leary B Kilburn L Hrebien S Garcia-Murillas I Beaney M Plasma ESR1 mutations and the treatment of estrogen receptor-positive advanced breast cancer. J Clin Oncol (2016) 34:2961–8. 10.1200/JCO.2016.67.3061 27269946

[24] Schiavon G Hrebien S Garcia-Murillas I Cutts RJ Pearson A Tarazona N Analysis of ESR1 mutation in circulating tumor DNA demonstrated evolution during therapy for metastatic breast cancer. Sci Transl Med (2015) 7:182 10.1126/scitranslmed.aac7551 PMC4998737 26560360

[25] Spoerke JM Gendreau S Walter K Qiu J Wilson TR Savage H Heterogeneity and clinical significance of ESR1 mutations in ER-positive metastatic breast cancer patients receiving fulvestrant. Nat Commun (2016) 7:11579. 10.1038/ncomms11579 27174596 PMC4869259

[26] Niu J Andres G Kramer K Kundrada MN Alvarez RH Klimant E Incidence and clinical significance of ESR1 mutations in heavily pretreated metastatic breast cancer patients. Onco Targets Ther (2015) 8:3223–328. 10.2147/OTT.S92443 26648736 PMC4648593

[27] Harrod A Fulton J Nguyen V Periyassami M Ramos-Garcia L Lai C Genomic modelling of the ESR1 Y537S mutation for evaluating function and new therapeutic approaches for metastatic breast cancer. Oncogene (2016):1–11. 10.1038/onc.2016.382 27748765 PMC5245767

[28] Fanning SW Mayne CG Dharmarajan V Carlson KE Martin TA Novick SJ Estrogen receptor alpha somatic mutations Y537S and D538G confer breast cancer endocrine resistance by stabilizing the activating function-2 binding conformation. Elife (2016) 5:e12792. 10.7554/eLife.12792 26836308 PMC4821807

[29] Ellis M. Overcoming endocrine therapy resistance by signal transduction inhibition. Oncologist (2004) 9:20–6. 10.1634/theoncologist.9-suppl_3-20 15163844

[30] Nagaraj G Ma C. Revisiting the estrogen receptor pathway and its role in endocrine therapy for postmenopausal women with estrogen receptor-positive metastatic breast cancer. Breast Cancer Res Treat (2015) 150:231–42. 10.1007/s10549-015-3316-4 25762475

[31] Ma C Bose R Ellis M Prognostic and predictive biomarkers of endocrine responsiveness for estrogen receptor positive breast cancer. In: Stearns V, editor. Novel Biomarkers in the Continuum of Breast Cancer, Advances in Experimental Medicine and Biology. London: Springer (2016). 882 p. 10.1007/978-3-319-22909-6_5 26987533

[32] Goss PE Ingle JN Pritchard KI Robert NJ Muss H Gralow J Extending aromatase-inhibitor adjuvant therapy to 10 years. N Engl J Med (2016) 375:209–19. 10.1056/NEJMoa1604700 27264120 PMC5024713

[33] Haber D Velculescu V. Blood-based analyses of cancer: circulating tumor cells and circulating tumor DNA. Cancer Discov (2014) 4:650–61. 10.1158/2159-8290.CD-13-1014 24801577 PMC4433544

[34] Dawson SJ Tsui DW Murtaza M Biggs H Rueda OM Chin SF Analysis of circulating tumor DNA to monitor metastatic breast cancer. N Engl J Med (2013) 368:1199–209. 10.1056/NEJMoa1213261 23484797

[35] Chu D Paoletti C Gersch C VanDenBerg DA Zabransky DJ Cochran RL ESR1 mutations in circulating plasma tumor DNA from metastatic breast cancer patients. Clin Cancer Res (2016) 22:993–9. 10.1158/1078-0432.CCR-15-0943 26261103 PMC4993201

[36] Sefrioui D Perdrix A Sarafan-Vasseur N Dolfus C Dujon A Picquenot JM Short report: monitoring ESR1 mutations by circulating tumor DNA in aromatase inhibitor resistant metastatic breast cancer. Int J Cancer (2015) 137:2513–9. 10.1002/ijc.29612 25994408

[37] Takeshita T Yamamoto Y Yamamoto-Ibusuki M Droplet digital polymerase chain reaction assay for screening of ESR1 mutations in 325 breast cancer specimens. Transl Res (2015) 166:540–53. 10.1016/j.trsl.2015.09.003 26434753

[38] Guttery DS Page K Hills A Woodley L Marchese SD Rghebi B Noninvasive detection of activating estrogen receptor 1 (ESR1) mutations in estrogen receptor-positive metastatic breast cancer. Clin Chem (2015) 61:974–82. 10.1373/clinchem.2015.238717 25979954

[39] Baselga J Campone M Piccart M Burris HA III Rugo HS Sahmoud T Everolimus in postmenopausal hormone-receptor-positive advanced breast cancer. N Engl J Med (2012) 366:520–9. 10.1056/NEJMoa1109653 22149876 PMC5705195

[40] Finn R Martin M Rugo H Jones S Im S Gelmon K Palbociclib and letrozole in advanced breast cancer. N Engl J Med (2016) 375:1926–36. 10.1056/NEJMoa1607303 27959613

[41] Turner NC Ro J André F Loi S Verma S Iwata H Palbociclib in hormone-receptor positive advanced breast cancer. N Engl J Med (2015) 373(3):209–19. 10.1056/NEJMoa1505270 26030518

[42] Hortobagyi G Stemmer S Burris H Yap YS Sonke GS Paluch-Shimon S Ribociclib as first-line therapy for HR-positive, advanced breast cancer. N Engl J Med (2016) 375(18):1738–48. 10.1056/NEJMoa1609709 27717303

[43] Johnston SR Kilburn LS Ellis P Dodwell D Cameron D Hayward L Fulvestrant plus anastrozole or placebo versus exemestane alone after progression on non-steroidal aromatase inhibitors in postmenopausal patients with hormone-receptor-positive locally advanced or metastatic breast cancer (SoFEA): a composite, multicentre, phase 3 randomised trial. Lancet Oncol (2013) 14:989–98. 10.1016/S1470-2045(13)70322-X 23902874

[44] Augusto L Sarafan-Vasseur N Perdrix A Beaussire L Delacour J Sefrioui D Prognostic and predictive value of circulating ESR1 mutations in metastatic breast cancer patients (mBC) progressing under aromatase inhibitor (AI) treatment. J Clin Oncol (2016) 34(Suppl; abstr 511).

[45] Finn RS Martin M Rugo HS Jones S Im SA Gelmon K Palbociclib and letrozole in advanced breast cancer. N Engl J Med (2016) 375:1925–36. 10.1056/NEJMoa1607303 27959613

[46] Finn RS Crown JP Lang I Boer K Bondarenko IM Kulyk SO The cyclin-dependent kinase 4/6 inhibitor palbociclib in combination with letrozole versus letrozole alone as fi rst-line treatment of oestrogen receptor-positive, HER2-negative, advanced breast cancer (PALOMA-1/TRIO-18): a randomised phase 2 study. Lancet Oncol (2015) 16:25–35. 10.1016/S1470-2045(14)71159-3 25524798

[47] Lauring J Wolff A Evolving role of the estrogen receptor as a predictive biomarker: ESR1 mutational status and endocrine resistance in breast cancer. J Clin Oncol (2016) 34(25):2950–2. 10.1200/JCO.2016.68.4720 27382095

[48] Dickler MN Tolaney SM Rugo HS Cortes J Dieras V Patt DA MONARCH1: results from a phase II study of abemaciclib, a CDK4 and CDK6 inhibitor, as monotherapy, in patients with HR+/HER2-breast cancer, after chemotherapy for advanced disease. J Clin Oncol (2016) 34(suppl; abstr 510).

[49] Pinkerton J Thomas S. Use of SERMs for treatment in postmenopausal women. J Steroid Biochem Mol Biol (2014) 142:142–54. 10.1016/j.jsbmb.2013.12.011 24373794

[50] Mayer I Phase I study of ARN-810, a novel selective estrogen receptor degrader, in postmenopausal women with locally advanced or metastatic estrogen receptor positive breast cancer [abstract]. CTRC-AACR San Antonio Breast Cancer Symposium OT3-2-07. San ANtonio (2013).

[51] Ladd B Mazzola AM Bihani T Lai Z Bradford J Collins M Effective combination therapies in preclinical endocrine resistant breast cancer models harboring ER mutations. Oncotarget (2016) 7(34):54120–36. 10.18632/oncotarget.10852 27472462 PMC5342331

[52] Weir HM Bradbury RH Lawson M Rabow AA Buttar D Callis RJ AZD9496: an oral estrogen receptor inhibitor that blocks the growth of ER-positive and ESR1-mutant breast tumors in preclinical models. Cancer Res (2016) 76:3307–18. 10.1158/0008-5472.CAN-15-2357 27020862

[53] Wang T Lonard DM Yu Y Chow DC Palzkill TG Wang J Bufalin is a potent small-molecule inhibitor of the steroid receptor co-activators SRC-3 and SRC-1. Cancer Res (2014) 74:1506–17. 10.1158/0008-5472.CAN-13-2939 24390736 PMC3947477

[54] Yu M Bardia A Aceto N Bersani F Madden MW Donaldson MC Cancer therapy: ex vivo culture of circulating breast tumor cells for individualized testing of drug susceptibility. Science (2014) 345:216–20. 10.1126/science.1253533 25013076 PMC4358808

[55] Yamamoto-Ibusuki M Arnedos M André F. Targeted therapies for ER+/HER2-metastatic breast cancer. BMC Cancer (2015) 13:137. 10.1186/s12916-015-0369-5 26059247 PMC4462184

[56] Chia YH Ellis MJ Ma CX. Neoadjuvant endocrine therapy in primary breast cancer: indications and use as a research tool. Br J Cancer (2010) 103:759–64. 10.1038/sj.bjc.6605845 20700118 PMC2966629

[57] Reinert T Ramalho S Gonçalves R Barrios C Graudenz M Bines J. Multidisciplinary approach to neoadjuvant endocrine therapy in breast cancer: a comprehensive review. Rev Bras Ginecol Obstet (2016) 38(12):615–22. 10.1055/s-0036-1597579 28002848 PMC10309433

[58] Miller CA Gindin Y Lu C Griffith OL Griffith M Shen D Aromatase inhibition remodels the clonal architecture of estrogen-receptor-positive breast cancers. Nat Commun (2016) 7:12498. 10.1038/ncomms12498 27502118 PMC4980485

[59] Bardia A Iafrate J Sundaresan T Younger J Nardi V Metastatic breast cancer with ESR1 mutation: clinical management considerations from the molecular and precision medicine (MAP) Tumor Board at Massachusetts General Hospital. Oncologist (2016) 21:1035–40. 10.1634/theoncologist.2016-0240 27551012 PMC5016066

[60] Ng C Schultheis A Bidard F Wigelt B Reis-Filho J Breast cancer genomics from microarrays to massively parallel sequencing: paradigms and new insights. J Natl Cancer Inst (2015) 107:djv015 10.1093/jnci/djv015 25713166

